# A Case of Triplets

**Published:** 1890-03

**Authors:** E. D. Chase

**Affiliations:** Galveston


### A CASE OF TRIPLETS.

BY E. D. CHASE, M. D., GALVESTON.

[Read at meeting of the Galveston County Medical Club at a regular meeting, February 10, 1890, and ordered to be printed in Daniel's Texas Medical Journal.]

I AM led to report this case, not that the labor was remarkable for length, severity, complications, or other point of obstetric interest excepting the number; not that it required any great amount of skill or tact to consummate it; but, partly from its rarity; occuring as it does but once in about 6000 labors; partly from the fact that it is the first case of the kind I can find recorded for this city; and partly to put it on record as statistics for those who love them, and as an item of curiosity for all.

I was summoned November 1, 1889, at 3 a. m., to see Mrs. J. W. H. White, age 27, who had been suffering more or less for five or six days, and with labor pains since midnight. This was her sixth confinement, the previous ones all single.

There was no record of plural births in any of her family, but the husband's sister had once given birth to twins.

I found a roomy pelvis, vagina well lubricated, the external os fully dilated, and through the bulging bag of membranes I could make out the head in the left occipito-posterior position. The pains were regular and seemed strong enough, but the progress of the labor was not as satisfactory as the circumstances seemed to demand. So at the next pain I sawed across the tense, bulging membrane with my finger nail when it ruptured, and about a pint of fluid came gushing out. A second pain almost immediately followed bringing the child, a girl, at 4 a. m. While engaged tying the cord, the mother informed me that something was wrong yet. A hasty examination revealed another bag of membranes at the os, and a little later I could make out a footling. As there was no longer any use for the bag of membranes, and the pains being strong, it was also ruptured. With another gush of water, and after a few pains—three or four—a boy was born at 4:25 o'clock.

Now was a good time to stop; so I was totally unprepared for the cheering anouncement "all isn't right yet, Doctor." This time I found a head in the right occipito-anterior position. The pains seemed now to have ceased, and she declared she would die as she "hadn't misery enough to bring it." A five grain quinine bi-sulphate pill and a small toddy with, after some little time, judicious manipulation of the uterus, and the pains recommenced. This time they were more severe and lasted longer, but seemed ineffectual. She wanted to give it up two or three times, but a little assurance that all was right and slight compression over the uterus, brought away the third, a boy, together with the placentæ and membranes, at 6 a. m. The child was enveloped in the placentæ and membranes, making comparatively a large body, and doubtless the cause of the longer, harder delivery. She was only a little tired, talked and joked as lively as ever. In all her previous labors she sat up within the hour and nursed her child, and usually got up and did her work after the seventh day. After seeing the babies properly attended to, I left the mother in charge of the nurse, to call again in the afternoon.

There was only about a quart of fluid expelled. Scarcely an ounce of blood escaped. There was a single and a double placenta united by membrane. The girl was attached to the single placenta, the two boys to the double one. There was only one sack for the boys, therefore no bag to rupture or waters to attend the delivery of the third child.

They were christened by the time I made my afternoon call, receiving the names, Mary Elizabeth, David and Joseph. Mary and David each weighed 2¾ pounds, and measured 16 inches in length, Joseph weighed 3 pounds and measured 17 inches. All were well formed, good featured, apparently healthy children, and seemingly as well fitted for the race of life as larger babes. Mary died of trismus nascentium on the 9th day, and Joseph of menengitis on the 13th. Little David still lives, is growing nicely, is healthy and hearty, and has had his "la grippe" with the rest.

### Correspondence.

### A WEEK OF PRACTICE IN MEXICO—THE DOCTORS PARADISE (?)

PRESIDIO DEL NORTE, MEXICO, Feb. 18, 1890.

*Daniel's Texas Medical Journal:*

